# Structural diversity of crustacean exoskeletons and its implications for biomimetics

**DOI:** 10.1098/rsfs.2023.0075

**Published:** 2024-04-12

**Authors:** Miloš Vittori

**Affiliations:** University of Ljubljana, Biotechnical Faculty, Department of Biology, Večna pot 111, 1000 Ljubljana, Slovenia

**Keywords:** biomechanics, biomineralization, arthropod, biomimetics, cuticle

## Abstract

The crustacean cuticle is a biological composite material consisting of chitin–protein fibres in a mineralized matrix. Recent research has revealed a surprising range of fibre architectures and mineral compositions of crustacean skeletal structures adapted to various mechanical demands. It is becoming increasingly clear that the organic fibres in the cuticle may be organized in patterns differing from the standard twisted plywood model. Observed fibre architectures in protruding skeletal structures include longitudinal and circular parallel fibre arrays. Skeletal minerals often include calcium phosphates in addition to calcium carbonates. Furthermore, skeletal properties are affected by protein cross-linking, which replaces mineralization as a stiffening mechanism in some structures. Several common structural motifs, such as the stiffening of the outer skeletal layers, the incorporation of non-mineralized cuticle in exposed structures, and interchanging layers of parallel fibres and the twisted plywood structure, can be identified in skeletal elements with similar functions. These evolutionary solutions have the potential for biomimetic applications, particularly as manufacturing technologies advance. To make use of this potential, we need to understand the processes behind the formation of the crustacean exoskeleton and determine which features are truly adaptive and worth mimicking.

## 1. Introduction

Crustaceans are a group of arthropods that encompasses more than 30 000 known species [1] inhabiting aquatic and terrestrial environments. A chitinous cuticle covers the body surface in a continuous sheet and forms the crustacean exoskeleton. The crustacean exoskeletal cuticle is a structural biological composite material that incorporates fibres of the polysaccharide chitin associated with proteins. These fibres may be embedded in a mineralized matrix in which calcium carbonate dominates. While the exoskeletal cuticle may be heavily mineralized, the proportion of organic fibres is considerable, often about a quarter of dry weight [2].

The diversity of crustaceans suggests that they have evolved various specializations of their exoskeletons that fit the diverse needs of their lifestyles. A crustacean's skeleton and its parts can be expected to be optimized by natural selection in agreement with their functions. If we identify features that perform well under certain mechanical demands, they can be mimicked in artificial systems.

**Figure 1.**
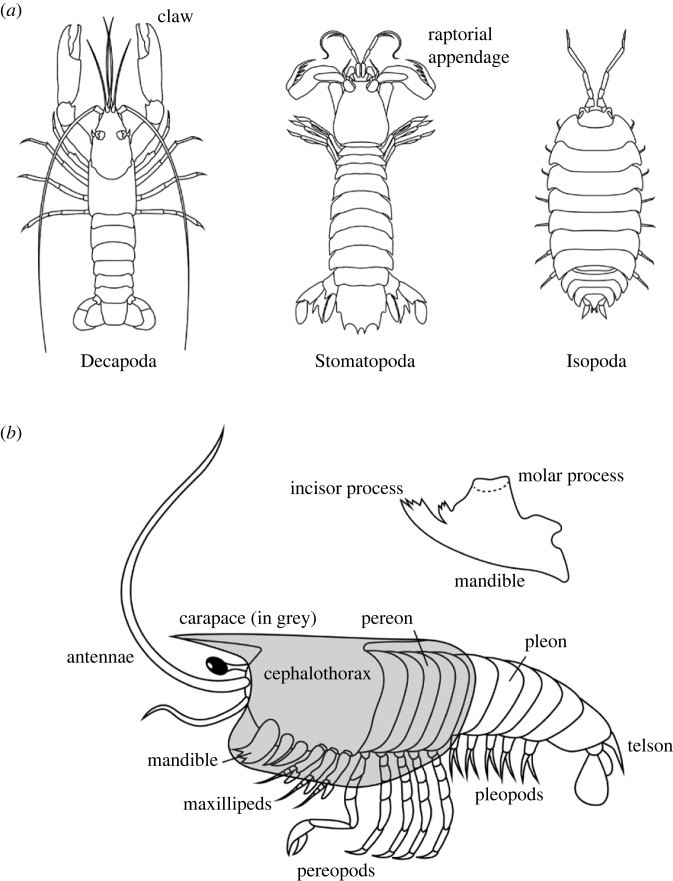
Malacostraca and their body structure. (*a*) The most studied groups of Malacostraca with regard to their exoskeletons. (*b*) A simplified drawing of crustacean morphology pointing out structures relevant to this text. The structure of the mandible is presented separately.

While the exoskeleton of numerous crustaceans has been studied in some aspects, studies of how its structure and composition affect its material properties have focused mainly on Malacostraca. Within this group of crustaceans, three orders stand out: Decapoda, including shrimps, lobsters and crabs; Stomatopoda or mantis shrimps; and Isopoda, the group that includes the terrestrial woodlice and numerous aquatic isopods (figure 1).

As various aspects of the skeleton in different crustacean groups are studied largely independently by biologists and materials scientists, this work is an attempt to bring some of the scattered information together and identify major structural motifs encountered across the skeletons of the most studied crustaceans. It also reviews recent advances in our understanding of the complexity of fibre architectures and surrounding minerals in crustacean skeletons and discusses the application of this knowledge in biomimetic designs.

## 2. The body of a crustacean

A brief overview of how crustacean bodies are constructed is necessary to discuss the properties of different parts of their skeletons (figure 1). The body of a crustacean consists of segments and these join together to form body regions, such as the head, the thorax and the abdomen. One or several anterior thoracic segments may fuse with the head to form a cephalothorax, while the remaining thoracic segments form another body region, the pereon. In Malacostraca, the posterior-most body region is called the pleon instead of the abdomen. Various regions of the body may bear appendages with a range of functions and names. A feature that distinguishes crustaceans from other extant arthropod groups are two pairs of antennae on their head, which also bears three pairs of mouthparts that are modified appendages. The first pair of mouthparts, the mandibles, are generally used for mastication, and they often have processes that aid this function. These are the incisor process at the tip of the mandible and the molar process near its base (figure 1). The appendages of the thorax are thoracopods, and they are referred to as pereopods if a pereon is present. The thoracic appendages are most often used for walking but may be modified for various modes of feeding: grasping, smashing, spearing, or filtering water. In cases when a cephalothorax is present, the maxillipeds are its appendages following the mouthparts. The abdomen typically lacks appendages, but in Malacostraca that possess a pleon, there may be numerous pleopods on this region. The cuticle on the dorsal side of the body segments forms plates called tergites, which are often particularly thick and stiff. Some crustaceans possess a carapace, a fold that extends from the dorsal surface and encloses the body either partly or entirely [3].

Stiff skeletal elements are linked by joints that enable their movement with respect to one another. However, crustacean joints are very different from those found in internal skeletons of vertebrates, as external surfaces of the skeleton are in contact. Even though two crustacean skeletal elements are linked by a mobile joint, the cuticle between them is continuous. The flexible region of the cuticle that enables the movement of a joint is the arthrodial membrane, which is thin and flexible [4,5]. The cuticle covering structures that participate in breathing and osmoregulation is also thin and non-mineralized [6]. The different functions that the cuticle performs in different skeletal elements calls for structural variation, which may be interesting to examine from the points of view of biomechanics and biomimetics.

## 3. Crustacean skeletons as biological composite materials

**Figure 2.**
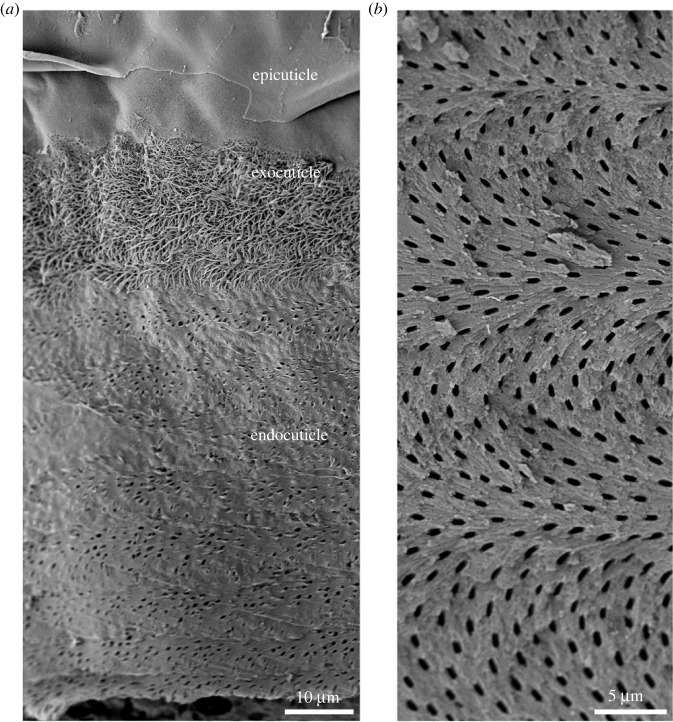
The organization of the crustacean cuticle. (*a*) A scanning electron micrograph of a decalcified tergite of the terrestrial isopod *Armadillidium* in oblique cross-section, showing the architecture of the organic matrix in the three major layers of the cuticle: the epicuticle, a thin sheet covering the external surface of the skeleton; the exocuticle, in which thick chitin–protein fibres are visible; and the endocuticle, which lacks thicker fibres. The surface of the tergite is at the top of the image. (*b*) An oblique fracture through the claw cuticle of the crab *Eriphia*. Fibres are arranged in the Bouligand pattern, shifting their orientation in sequential planes. The images were obtained as described in Vittori *et al*. [7] (*a*) and Vittori *et al*. [8] (*b*).

A general description of the crustacean cuticle was initially obtained from dorsal plates and the carapace of Decapoda, and these are good examples of its basic architecture. As with other invertebrate exoskeletons, the cuticle is not a homogeneous covering of the body. Based on differences in structure, composition and the time of deposition, three main layers can be distinguished in the cuticle. These layers are the epicuticle, the exocuticle and the endocuticle [6] (figure 2).

The epicuticle is very thin and forms the outer surface of the skeleton. It can be sculpted into diverse scales and sensory structures [9]. Generally, the epicuticle is considered to consist predominantly of proteins and lipids, but there have been clear demonstrations of mineral [4,6,10], and chitin may be present at least in small amounts, as demonstrated by lectin labelling [11]. Underneath the epicuticle lies the exocuticle, a thicker layer that contains chitin–protein fibres and is often the most heavily mineralized layer of the cuticle. In most cases, the mineral in this layer is in crystalline form, in the case of calcium carbonate in the form of calcite. [6,12]. The innermost layer is the endocuticle, which is typically the thickest layer. It consists of numerous layers of chitin–protein fibres and is mineralized, often with amorphous calcium carbonate and small amounts of calcium phosphate [6,12]. In some species, the innermost layers are free of mineral. In these cases, they may be referred to as the membranous layer [6].

In the endocuticle and the exocuticle, chitin–protein fibres shift their orientation by a certain angle in sequential planes, creating a helicoidal pattern. These shifts in orientation are visible in cross-sections of the cuticle as lamellae, each representing a 180° turn of fibre orientation (figure 2). This fibre organization, universally present among arthropods, has been referred to as the twisted plywood structure or the Bouligand structure [13]. It provides the cuticle with isotropic mechanical properties in the plane of the cuticle [14]. The number and thickness of lamellae in the cuticle differ between layers of the cuticle [15], between regions of the body [6] and between species [16,17]. It has been shown that as crustaceans grow, their cuticles most often become thicker [8,16]. This thickening is accompanied by a thickening of the lamellae, suggesting that in thicker cuticles, fibres in sequential layers shift their orientation more gradually.

In decapods, the exocuticle often consists of thinner lamellae than the endocuticle and this is associated with increased stiffness [6,15], even in some cases when it is not accompanied by an increase in calcium concentration [18]. However, an overview of the cuticular structure across the Malacostraca reveals that while thinner lamellae are present in the exocuticle of benthic decapods, this is hardly ever the case in other crustacean groups [16,17].

A distinction has been made between chitin–protein fibrils and chitin–protein fibres. Fibrils have a diameter of only 3–5 nm and a length of a few hundred nanometres, while fibres are bundles of these fibrils linked by proteins [6,19]. In some cases, it has been proposed that certain cuticular layers, such as the endocuticle of isopods and the membranous layer of crabs, do not contain defined fibres, but strings of individual fibrils instead, as the thickness of observed structures matches that of individual fibrils [10]. While the distinction between the two structures is important, the term fibre will be used throughout this text for simplicity. We may also consider that the fibrils may interconnect without forming defined fibres [20].

A nearly universal feature of crustacean cuticles are pore canals, which run across the cuticle (figure 2). They are often not empty spaces, but contain fibres as well as mineral, which may differ from the mineral in the surrounding cuticle [6,10].

## 4. The process of cuticle formation

The cuticle does not grow continuously. Instead, crustaceans deposit a new skeleton and shed the old one periodically, allowing them to grow, develop and regenerate. Replacing the old cuticle with a new one by moulting dictates many aspects of crustacean biology, as well as the structure of the crustacean exoskeleton. Many crustaceans undergo periodic moulting even after they reach sexual maturity. In these cases, crustaceans deposit new skeletons regularly and often frequently, sometimes with only weeks between moults [21,22].

It is important to be familiar with the processes of cuticle formation and mineralization, as they can be expected to represent evolutionary constraints on the skeletal structure and composition. Furthermore, some characteristics of the skeleton may be direct consequences of how it is formed without having any mechanical adaptive significance, an important aspect that should be considered when interpreting the functions of skeletal features.

A new cuticle is formed in a sequence of processes taking place before and after the shedding of the old cuticle. During preparation for the moult, the epidermis first detaches from the old cuticle and deposits the epicuticle. This is followed by the deposition of the exocuticle, which also generally takes place before the moult. The old cuticle is then shed and the two deposited layers assume their final shape. The remaining layer, the endocuticle, is usually deposited after the moult [6]. There are, however, exceptions to this generalized description, and we will examine some of them.

Depositing a new skeleton that assumes its ultimate shape after the moult necessitates it to be pliant before the old skeleton is shed and hardened afterwards, with sclerotization and mineral deposition taking place after the moult [23,24]. The shifting of calcium from the old cuticle to transient mineral deposits in various parts of the body, and to the newly formed cuticle has been studied in isopods at the level of the entire body using micro-CT [11,25], and at high resolution using a combination of Raman imaging and scanning electron microscopy [24]. The mineralization of the newly deposited cuticle in isopods is fast: the exocuticle is mineralized within an hour after the old cuticle is shed in isopods [24]. This process likely takes much longer in crabs, between hours and days [23,26]. In this case, sclerotization, the cross-linking of cuticular proteins, can stiffen the new cuticle while mineralization is taking place [26].

The epidermal cells of crustaceans can shape the cuticle in complex forms, which is remarkable if we consider that these cells must alter the chemical composition of the cuticle and deposit mineral after the organic components. The precise mechanism of chitin fibre formation is not understood, but past observations using electron microscopy indicate that they are formed at some distance from the epithelial cell surface. This, along with the ability of short chitin fibrils in suspension to spontaneously form chains that self-organize to form the Bouligand pattern [27], has led to a model of chitin deposition that proposes a self-organization of fibrils and fibres, with the characteristics of the resulting fibrous structure determined by cuticular proteins [6].

While it might seem intuitive for these processes to take place synchronously across the entire epidermis, it has been shown that the epicuticle and exocuticle of some skeletal structures can form earlier than in others [6]. This is perhaps most obvious in isopods, which are characterized by the biphasic moult, shedding the cuticle of the posterior body segments first and anterior ones later [28]. However, there are also cases in which the typical sequence of layer deposition does not occur, and the entire cuticle is deposited before the moult. A game-changing discovery was made in the mandibles of isopods. The analysis of the formation of the mandibular incisor processes in the terrestrial isopod *Porcellio scaber* demonstrated that they are mineralized before the moult [25,29]. Furthermore, the strongly mineralized layer of the incisor cuticle is the endocuticle, demonstrating that the entire cuticle of the incisor process is deposited before the moult by a population of structurally different epidermal cells [29]. The complete formation of a new cuticle before the moult is also known from decapod gills [30], the cuticle of which is not mineralized. We can therefore conclude that the timeline of cuticle deposition differs between skeletal elements and body regions and that the division of the cuticle into layers deposited before and after the moult is not universal.

## 5. A plot twist in twisted plywood

The Bouligand pattern is a common way in which arthropods organize fibres in their skeletons, but is it the only way? Apparently not, as recent studies demonstrate that the fibre orientation is tailored in accordance with the forces a skeletal element withstands. The first example we can investigate was identified in the terrestrial isopod *P. scaber*.

**Figure 3.**
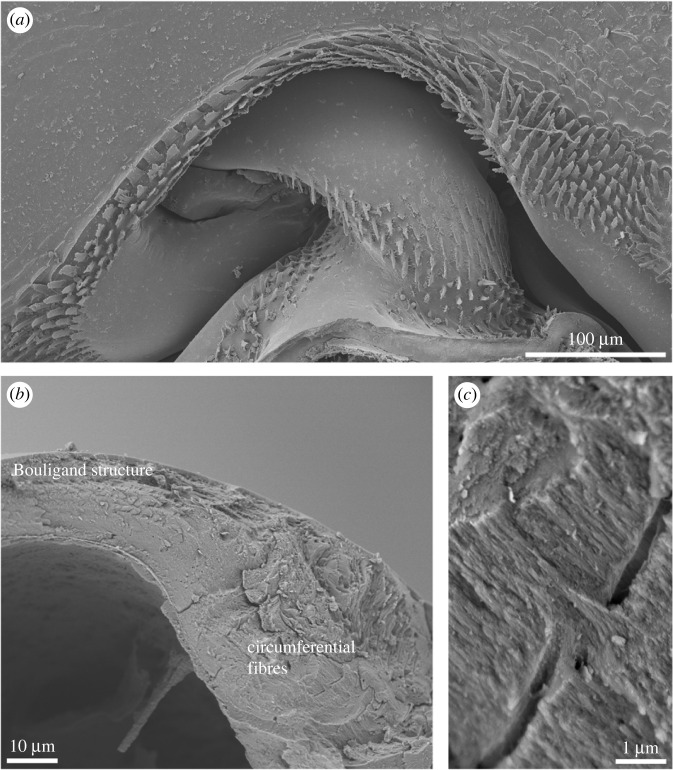
Parallel fibres in the joint head of a terrestrial isopod leg. (*a*) A scanning electron micrograph of the joint on the leg basis in *Porcellio*. (*b*) A fractured joint head displaying the Bouligand structure in the central region and bundles of circumferentially oriented fibres near the edge. (*c*) A higher magnification scanning electron micrograph of the circumferential fibres, showing their parallel arrangement. Images were obtained as detailed in Vittori *et al*. [8].

Isopods have seven similar pairs of legs. The highly mobile joint at the base of their walking legs consists of a rounded head that fits into a socket (figure 3). While the cuticle of the central region of the joint head is organized similarly to the tergal cuticle of isopods, its edge region is not. The chitin–protein fibres are oriented in parallel along the edge of the joint head, but only in the inner cuticular layer, the endocuticle [4].

In *P. scaber*, the alignment of fibres in a single direction is even more pronounced in the claws of the walking legs. The organic fibres in the leg cuticle are organized in the typical Bouligand pattern but they all align in the axial direction in the claw cuticle [31]. This is also the direction of loading when the animal stands or walks on the tips of its claws [5].

However, a simple alignment of fibres is not the only deviation from the Bouligand structure found in crustaceans. While some mantis shrimps spear their prey, others have smashing appendages that allow them to break the shells of hard-bodied prey. Both groups use the second pair of thoracic appendages for prey capture. The most distal article of this appendage, the dactylus, bears the impact surface in smashers, and it was studied in detail in *Odontodactylus scyllarus* [32]. Several regions differing in fibre organization were identified in its smashing limb cuticle. The striated region that is positioned laterally on the dactylus contains circumferentially oriented parallel fibres. The inner layers of the cuticle form the periodic region with fibres organized in accordance with the Bouligand pattern. In the impact region that forms the smashing limb surface, the fibres form a modified, undulating Bouligand pattern, appearing as a herringbone pattern in cross-section [32,33]. The sinusoidal undulations of stacked layers of fibres result in a large proportion of them running perpendicularly to the impact surface. As predicted by finite-element modelling, this organization redistributes stresses on impact, limiting their localization and thus preventing failure [33].

Layers of parallel fibres are not limited to elongated skeletal elements in isopods but are also found in certain regions of their tergites. In the desert isopod *Hemilepistus reaumuri*, the endocuticle forms thick layers of transversely oriented parallel fibres near the anterior and posterior edges of the tergites. The parallel organization of the fibres in this case is believed to increase the bending strength of the tergite [34].

**Figure 4.**
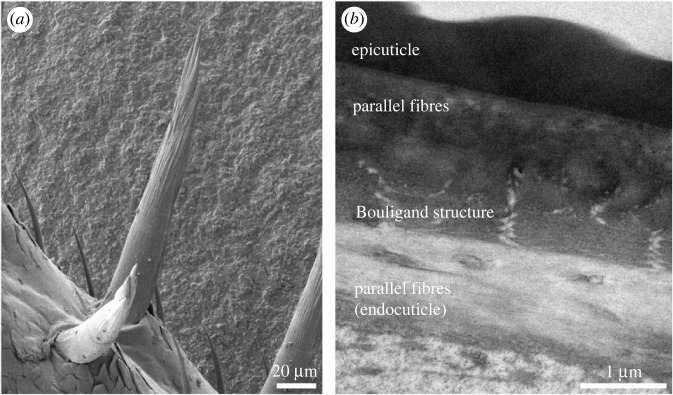
Chitin–protein fibre organization in the sensory spines of the terrestrial isopod *Porcellio*. (*a*) A scanning electron micrograph of sensory spines on the walking leg. (*b*) A transmission electron micrograph of the spine cuticle in longitudinal section. While the fibres in the outer exocuticle are parallel, those in the inner exocuticle form the Bouligand structure. In the endocuticle, fibres are again parallel.

The utilization of different fibre arrangements can also be taken to greater complexity, with interchanging layers of twisted plywood arrangement and parallel fibres. The legs of *P. scaber* do not only bear claws, but large sensory spines as well (figure 4). In the spines, the fibres of the endocuticle are parallel and directed along the spine's length, while the exocuticle forms an inner layer featuring the Bouligand pattern and an outer layer of parallel fibres, again directed along the axis of the spine [35]. A somewhat similar arrangement of fibres was reported in mantis shrimps of the spearing type, the raptorial appendages of which bear long spikes [36,37]. In this case, the inner layer of the exocuticle forms the Bouligand pattern and the endocuticle is divided into a thick outer layer of parallel fibres and a thinner inner layer with fibres organized in the Bouligand pattern. This is the case in the appendage bearing the spikes [36] as well as in the spikes themselves [37]. A very similar arrangement of layers with contrasting fibre orientations was described in the spikes on the mantis shrimp telson [38].

In the incisor process of *P. scaber* mandibles, the fibre organization is even more complex. The external layers of the cuticle forming the tip of the incisor process consist of an external layer of longitudinally oriented fibres, an intermediate layer of circumferentially oriented fibres, and an inner layer of longitudinally aligned fibres, while the even deeper endocuticle is organized in the Bouligand pattern. Further towards the base of the incisor, the external cuticular layer combines longitudinally oriented fibres with fibres oriented towards the surface, while the endocuticle assumes a more complex architecture, comprising an external layer of circumferentially oriented fibres, a thick middle layer of longitudinally aligned fibres, and an inner layer with twisted plywood structure. The cuticle forming the body of the mandible conforms to the classical Bouligand organization that characterizes the tergites of this species [39].

The preferential orientation of the organic fibres in the longitudinal direction of elongated skeletal elements results in a mechanical anisotropy of the cuticle that contrasts the isotropic mechanical properties of the Bouligand structure. This has been demonstrated in isopods [39,40] as well as in mantis shrimps [14].

In all these cases, the fibre orientation is not the only thing that deviates from the generalized description of the cuticle. The mineral surrounding the fibres is also unorthodox or may be lacking altogether, and we can examine this in more detail.

## 6. Alternatives to calcium carbonate mineralization

**Figure 5.**
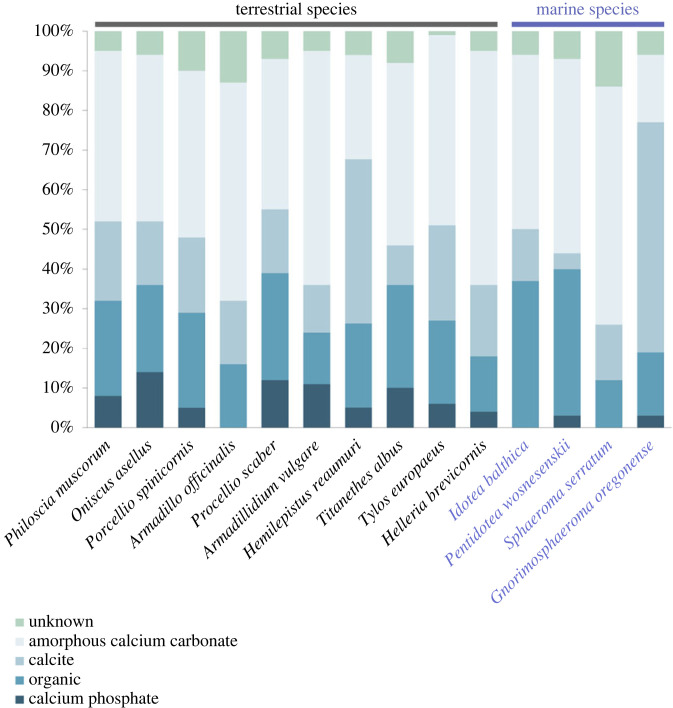
Proportions of tergal cuticle dry weight attributed to its components in different isopod species. Data were combined from [2,41–44].

Calcium carbonate is the stereotypical mineral in crustacean exoskeletons, and most crustaceans truly incorporate a lot of it. Much of this mineral may be in the form of calcite, but amorphous calcium carbonate often dominates (figure 5). Nevertheless, minor amounts of calcium phosphate are present as well (figure 5). In terrestrial isopods, amorphous calcium phosphate accounts for up to 20% of the mineral in their tergites [2].

There are skeletal elements, however, in which calcium phosphate dominates. The mandibles of the crayfish *Cherax quadricarinatus*, as in many other crustaceans, consist of a base, a molar process and an incisor process (figure 1). The molar process in this species is more heavily mineralized than the rest of the mandible. The surface layers of the cuticle forming the molar process were found to contain calcium phosphate in the form of fluorapatite, with the appearance of the mineral particles as well as the hardness and stiffness of the cuticle resembling that of vertebrate enamel [45,46]. The body cuticle of *C. quadricarinatus* had been studied in detail previously, and this feature is not its general characteristic [47]. In the case of the freshwater shrimp *Macrobrachium rosenbergii*, the incisor process of the mandible is also mineralized predominantly with calcium phosphate. In this case, apatite is present along the central axis of the incisor process [45,46].

Another case of apatite presence in the cuticle came from mantis shrimps. Among smashers, analysis of the smashing limb dactlyus in the genus *Gonodactylus* revealed that the surface layers of its cuticle contain mineral with a very high phosphorus to calcium ratio, indicating that it is mineralized predominantly with calcium phosphate. Corresponding measurements of hardness using nanoindentation demonstrated that these regions are also much harder than the underlying cuticular layers [48]. Subsequent research on the smasher *O. scyllarus* demonstrated that the outer layers of the club are heavily mineralized, extremely stiff and contain apatite. The chitin fibres and the apatite crystals are oriented towards the surface, in the direction of the impact force, further strengthening the structure. Underlying cuticular layers contain a smaller proportion of mineral, which is an amorphous mixture of calcium carbonate and calcium phosphate [32,36].

The use of apatite in raptorial appendages is not an exclusive characteristic of smashers. Another instance of apatite use was identified in mantis shrimps of the spearing type. In these, the external layers of the exocuticle of their raptorial appendages are heavily mineralized with apatite, while deeper layers contain amorphous calcium phosphate and calcium carbonate, as observed in corresponding layers of smashers [36,37]. Apparently, the strong degree of mineralization of the exocuticle with apatite is a general character of stomatopod dactyli that resist great impact forces during the spearing or smashing of prey.

Mineralization with calcium phosphate turned out to be widespread in the mandibles of Malacostraca [49]. In several representatives of this group, the mineral in the mandibles is apatite. This shows that the ability to deposit relatively pure calcium phosphate in certain regions of the skeleton is a general feature of Malacostraca and was likely present in ancestral crustaceans. In the case of isopods, however, another form of calcium phosphate is employed in the mandibles. The incisor process of *P. scaber* is mineralized with calcium phosphate, but in amorphous form. Mineralization with amorphous calcium phosphate results in lower stiffness and hardness of the incisor process than the calcite-mineralized cuticle of the mandible base [39]. This results in a gradient of hardness and stiffness, with non-mineralized cuticle at the tip of the incisor process, followed by cuticle mineralized with amorphous calcium phosphate in the middle and cuticle mineralized with calcite and amorphous calcium carbonate at the base of the incisor process, each sequential region being stiffer and harder [39].

A further example of a structure mineralized with amorphous calcium phosphate are the dactyli of terrestrial isopods. The claws on the isopod walking legs are covered by a thick layer of non-mineralized cuticle. The inner layers of the claw cuticle, on the other hand, are mineralized entirely with amorphous calcium phosphate. As the organic fibres in the claw are aligned in the axial direction, this affects the arrangement of mineral between them, resulting in a continuous, fibrous mineral structure oriented in the axial direction as well [31,40]. As a result of fibre arrangement, both the non-mineralized external layers and the mineralized internal layers of the cuticle are also mechanically anisotropic and much stiffer in the axial direction [40]. In sensory spines of isopod legs, which are also elongated and exposed structures, both the exocuticle and the endocuticle are mineralized with amorphous calcium phosphate [35].

The orientation of the c-axes of mineral crystals is often also aligned with the direction of force, like the chitin–protein fibres. This is the case in impact surfaces of mantis shrimps in both spearers and smashers, in which apatite crystals are oriented with their c-axes towards the surface [32,36]. The same is true for apatite crystals in the crayfish mandible, which are oriented towards the surface of the molar [46] and in the shrimp incisor process, where they are directed along its long axis [45,46]. By contrast, the c-axis of calcite crystals is largely directed parallel to the surface in isopod tergites [50]. This makes sense if we consider that tergites largely resist bending.

## 7. Durable cuticle without mineral

Clearly, certain regions of the cuticle must be flexible and therefore expected not to be mineralized. Obvious examples are arthrodial membranes and the gill cuticle [6]. By contrast, areas of the skeleton that are exposed to wear and heavy loading, such as the tips of claws and mandibles, can be expected to consist of particularly hard, stiff cuticle with a high proportion of crystalline mineral. In many cases, this is true [32,46,51]. This makes exposed skeletal elements with soft surfaces particularly interesting, and as it turns out, they are not rare.

A study of the tips of claws in grapsid crabs, used for manipulating food items and not for crushing, demonstrated that they are not mineralized, but contain elevated amounts of bromine [52]. This cuticle is not as stiff or hard as the mineralized cuticle of the claw base but has much greater fracture resistance [53]. The thick surface layer of the claw in terrestrial isopods turned out to not be mineralized at all, but heavily brominated, just as in grapsids [31,40]. As demonstrated for the isopod *Ligia pallasii* and the grapsid crabs, the non-mineralized, brominated cuticle of the claw has remarkably high hardness and modulus values considering that it is not mineralized [40,52].

In amphipods, a group of Malacostraca closely related to isopods, very similar features were demonstrated in the incisors of the mandibles in the freshwater amphipod *Acanthogammarus grewingkii* [54]. Elevated amounts of bromine were also demonstrated in spines of the gastric mill and setae on the mouthparts in the deep-sea amphipod *Hirondellea gigas* [55]. In a previous work on these amphipods, bromine was mistaken for aluminium [56] due to partial overlap of the K-peaks of aluminium and bromine in energy-dispersive X-ray spectra.

In these non-calcified cuticular regions, the mechanism behind their stiffening is the cross-linking of proteins: sclerotization. Brominated tyrosine compounds have been identified in the crustacean cuticle and suggested as cross-links involved in sclerotization [57]. This can explain the elevated amounts of bromine in exposed skeletal elements, such as isopod and crab claws. The bromine in these cases indicates a high degree of sclerotization of these structures. As demonstrated by X-ray absorption spectroscopy, the bromine in non-mineralized cuticle of crabs is indeed likely bound to phenolic amino acids [53,58]. As in cases listed above, the spines on the claws of crayfish and the tips of dactyli on walking legs of crabs are not calcified and are instead heavily sclerotized, as determined by applying a range of chemical treatments [59].

While it is generally accepted that the crustacean cuticle undergoes sclerotization after the moult, less is known about this process than in insects, in which biochemical pathways, enzymes and genes involved have been studied in detail [60]. These aspects have not been examined in crustaceans. The formation of phenolic cross-links by phenol oxidases has been proposed as a mechanism of crustacean cuticle sclerotization [6] and diphenols have been demonstrated in crustacean cuticle histochemically and suggested as possible cross-links [61].

## 8. Common themes

The skeletons of crustaceans are complex and diverse but there are common principles that can help us deal with this complexity. Considering the findings of published studies of various skeletal elements in different crustaceans, we can propose some generalizations:

— The surface layers of the skeleton are stiff and supported by softer, flexible layers containing less mineral that is in amorphous form. This is the case in the tergites of isopods and the carapace of decapods [12,15,62], but it is even more obvious in skeletal elements exposed to impacts and heavy loads during feeding, such as the mantis shrimp smashing and spearing appendages and the crayfish mandible, all of which have apatite near the surface and amorphous calcium minerals in deeper cuticular layers [32,36,46]. Hardness measurements across the cuticle of denticles on the claws of several crab species showed similar properties: hard external layers and softer inner layers of the cuticle forming these structures [51]. In these cases, it has been proposed that this combination effectively toughens the skeleton. The stiff surface prevents penetration, while the flexible internal layers dissipate energy and provide toughness as well as limit crack propagation [32,63]. The arrangement of stiff external layers, which resist compression well, and inner flexible layers, which are suitable to resist tension, was also observed in the cuticle of a region of mantis shrimp raptorial appendages that functions as a spring [64].
— Structures that withstand predictable loads incorporate parallel fibres oriented in the direction of the predominant loading. This was demonstrated in the isopod claws in which chitin–protein fibres are oriented longitudinally [5,31] as well as in the incisor processes of shrimps and isopods [39,45,46]. In surface layers of structures that are exposed to impacts and pressure, chitin–protein fibres are oriented towards the surface. This is the case in the crayfish molar process [46], the heavily mineralized denticles of crab claws [51], and in non-mineralized cuticle in the tips of the crab claws [52]. This principle can also be applied to the parallel fibres in the edges of isopod tergites [34].
— Elongated structures exposed to unpredictable loads combine interchanging layers of longitudinal, parallel fibres and layers organized in the Bouligand pattern. Such architecture has been identified in the mantis shrimp spikes [36–38], the isopod sensory spines [35] and the isopod incisor processes [39].
— Dome-shaped structures that withstand heavy loads without deforming are reinforced with bands of circumferential fibres around their perimeter. Such bands of parallel fibres were identified in the mantis shrimp smashing appendages [32], the isopod joints [4] and the isopod incisor processes [39].

**Figure 6.**
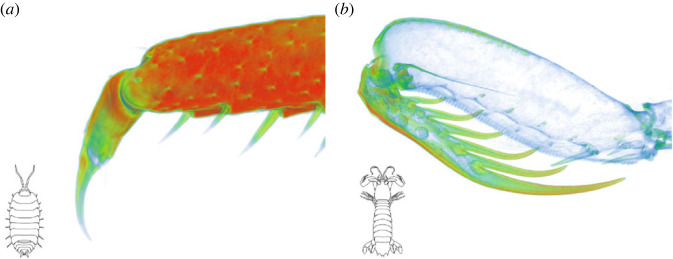
Two opposing approaches to constructing tough elongated structures, visualized by mapping X-ray opacity in micro-CT reconstructions. (*a*) The claw on a walking leg of the terrestrial isopod *Porcellio* is mineralized in its inner layers (red, green), while the surface and the tip of the claw are purely organic (blue). (*b*) The dactylus and its spikes in the mantis shrimp *Squilla* are much more heavily mineralized than the rest of its raptorial appendage. Both structures rely on calcium phosphate; it is in its flexible amorphous form in the claw of *Porcellio*, while *Squilla* also incorporates the hard and stiff apatite. Both approaches to making protruding structures durable can be found in various exposed skeletal elements in other crustacean groups, such as decapods. Images were obtained from ethanol-fixed specimens using a NeoScan N80 micro-CT device and processed using Dragonfly software (Object Research Systems). The leg of *Porcellio* was air-dried before imaging.

Interestingly, there are two contrasting strategies of constructing durable, protruding skeletal elements (figure 6). In some cases, the external surface is extremely hard and stiff, with a high proportion of mineral in crystalline form. This is the case for raptorial appendages of stomatopods, crayfish molar processes and denticles of certain crabs. By contrast, there other cases where such structures are covered by non-mineralized, sclerotized cuticle and only the inner layers of the cuticle are mineralized. This is a characteristic of isopod incisor processes and claws, amphipod mouthparts, and tips of claws in some crabs. Both approaches can result in less wear under certain conditions [14,53] and it is possible that different uses of a structure favour a different strategy. Mantis shrimp raptorial appendages penetrate prey, necessitating a hard surface, whereas the claws of terrestrial isopods do not and may instead reduce wear by being covered in flexible cuticle. Crabs apparently switch between both strategies. Hard claws of some crabs are used to crush and cut their food while the soft claw tips of others are used for scraping.

## 9. Functions and adaptations

To meaningfully interpret the functional value of different structural features of crustacean exoskeletons, a good knowledge of cuticular structure, processes of cuticular deposition and evolutionary histories of crustaceans in question is necessary. While it is valuable to discuss and interpret the possible adaptive significance and function of a feature when it is described and this should not be discouraged, we should be reserved when accepting such interpretations, discussions and suggestions as facts before demonstrating their plausibility.

Not all observed features of skeletons are due to mechanical adaptations. Some may result from processes of development and cuticular deposition. For example, certain features, such as the thickness of the cuticle, the thickness of the cuticular lamellae, and the relative contribution of different cuticular layers change as crustaceans grow [8,16]. When making comparisons between species and deriving supposed adaptations to their different lifestyles, this should be considered.

**Figure 7.**
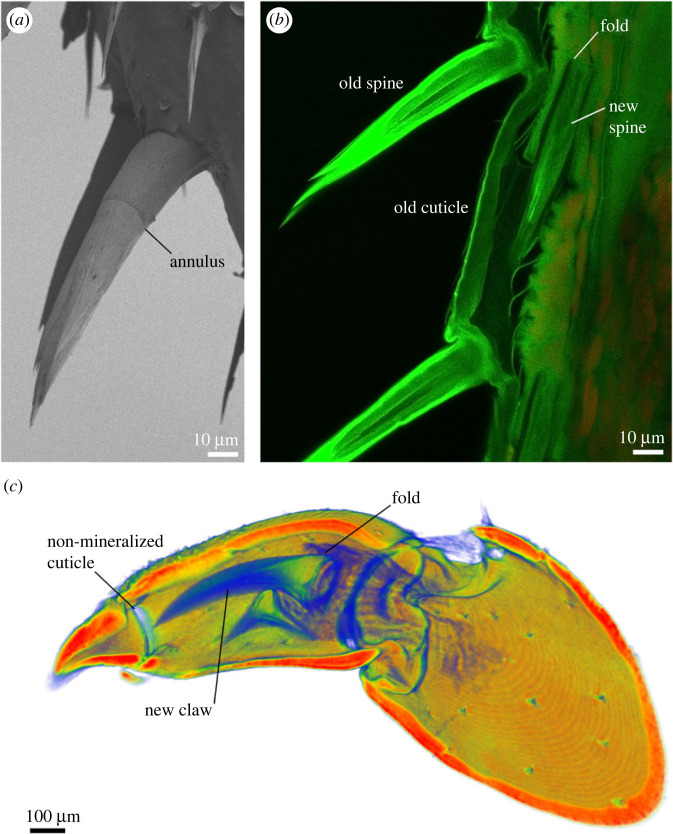
The formation of skeletal elements affects their structure. (*a*) A scanning electron micrograph of a sensory spine on an isopod leg with a clearly visible annulus. The image was obtained as described in Vittori *et al*. [35]. (*b*) Formation of the spines during preparation for moult in an optical section obtained with confocal microscopy. As new sensory spines are constructed, they are folded in an epidermal pocket. As they extend after moult, the position of the fold remains visible as the annulus. The leg was processed and imaged as described in Vittori *et al*. [65] using a Leica Stellaris 8 confocal microscope. (*c*) Formation of claws on the legs in the isopod *Ligia* visualized using micro-CT. The image was obtained as described in Vittori *et al*. [40] using a NeoScan N80 micro-CT device. Just like sensory spines, the claw forms in an invagination that remains visible as a ring of non-mineralized cuticle when the claw is extended. Is this simply the result of the claw's formation or does it perform a mechanical function?

An example of how the formation of the skeleton affects its structure is the annulus, a universal feature of crustacean setae (figure 7). During preparation for the moult, the new skeleton is partly deposited underneath the old one. The simple way of fitting an equal or larger new cuticular surface within the old one is by folding it, and this occurs with new setae (figure 7). The point where the setal cuticle is folded as the basal region of the seta forms results in the appearance of an annulus as the seta is extended after the moult [9] (figure 7). The annulus is therefore the result of the way in which setae are formed without necessarily having a mechanical function.

The case of the annulus is straightforward but other features can be more difficult to interpret. The claw on the legs of the isopod *Ligia* has a region of non-calcified cuticle at its base [40]. While this might have a mechanical function, the claw is folded during deposition and extends after the moult, just like the sensory spines (figure 7). The region of non-calcified cuticle could thus be a consequence of this mechanism of deposition.

The presence of amorphous minerals in the inner layers of the cuticle and crystalline phases in the external layers has often been interpreted mechanically as an adaptive feature that increases fracture resistance. The strengthening of a material by this arrangement of stiffer and more pliant layers has been demonstrated experimentally [14]. An alternative interpretation has also been proposed, however: amorphous minerals in the inner layers of the cuticle are much more soluble than crystalline ones, and it has been shown that the mineral in these layers is resorbed before the moult [12,24,46]. Are the amorphous minerals in inner cuticular layers there because they offer a mechanical advantage or because they are easily mobilized from the skeleton before it is replaced? In this case, we do not need to view these possibilities as alternative hypotheses. There is no reason not to resorb amorphous mineral and store it before the moult even if it has a mechanical function. Both effects can be beneficial, but it is more difficult to establish which had been selected for. The question of function and the selection pressures that led to the evolution of certain features are often the most difficult aspects of a biological problem to address experimentally.

Approaches to establishing the adaptive value of a structure include comparing structural features across phylogenies; determining how well a certain feature performs a function in comparison to alternatives; and experimentally demonstrating how the presence of a feature affects fitness [66]. Not all these approaches are always feasible, but many are, allowing hypotheses on functional and adaptive values of traits to be tested. The effect of fibre organization on crack propagation in the exoskeleton has been examined by nanoindentation followed by scanning electron microscopy and by *in situ* experiments using transmission electron microscopy [33]. By examining the failure of the mantis shrimp's spike cuticle and synthetic approximations of the spike cuticle, it has been demonstrated that a layer of Bouligand structure between the hard exterior and the flexible interior effectively limits crack propagation and failure of the spine [14].

Ultimately, it can be difficult to untangle which features are mechanical adaptations, which are ancestral traits, and which result from the mechanisms of cuticle deposition. Furthermore, these causal agents are not entirely separate from one another. If something demonstrably works mechanically, the evolutionary scenario that led to this feature may seem irrelevant. It is never irrelevant to biologists, as evolutionary history and the resulting functioning of living systems are the interest of our research. Yet it may also be important for biomimetics, as good ways of identifying likely adaptations allow us to better identify which features are worth mimicking.

## 10. Crustacean skeletons and biomimetics

The knowledge of how mechanical problems are solved in crustacean exoskeletons can be applied to the design of biomimetic composite materials and structures. This has been explored when it comes to the classical Bouligand structure of fibres embedded in a matrix [67–69] as well as the formation of helicoidal fibrous composites via self-organization [27]. The Bouligand structure was demonstrated to increase the impact resistance of synthetic materials by preventing the delamination of fibre layers and increasing energy dissipation by redirecting cracks that form on impact [68]. Furthermore, laminated sheets with greater changes in angle between layers near the loaded surface than near the opposite surface, an arrangement observed in mantis shrimp and crab cuticles, were shown to resist impact better [69]. The development of manufacturing methods enables a relatively straightforward transfer of crustacean skeletal features to artificial designs. Synthetic fibres can be deposited with controlled orientation and the properties of the surrounding matrix can be made less homogeneous than previously. Available approaches to producing materials that mimic the characteristics of the crustacean cuticle include three-dimensional printing, cholesteric self-assembly and electro-spinning [70,71].

Here, we can focus on some examples of crustacean bioinspired structures moving beyond the twisted plywood model. This has likely been explored best in replicating different elements of mantis shrimp skeletons. By three-dimensional printing of polymer fibres subsequently embedded in a resin matrix, bioinspired composite objects were produced that incorporated a Bouligand structure core and circumferential fibres arranged either in helices or in rings. The inspiration for this fibre organizations came from the mantis shrimp smashing appendage, which incorporates circumferentially oriented fibres, and from pore canals in crab exoskeletons. Mechanical testing and finite-element modelling were then used to examine the effects of such reinforcements on the biomimetic structures, demonstrating that helicoidally and circumferentially reinforced structures resisted shear better [72]. Multimaterial three-dimensional printing of fibres in a softer matrix was used to synthetically recreate the fibre arrangement of mantis shrimp telson spikes [38]. This manufacturing approach also enabled the construction of biomimetic structures that combine the various fibre orientations in different cuticular layers of the mantis shrimp spike with the differences in stiffness between them. Objects produced with these combined properties were much tougher than simpler designs with exclusively parallel fibres [14]. The impact region of the mantis shrimp smashing appendage was mimicked by three-dimensional printing of a composite consisting of polymer fibres in an elastic matrix. The resulting material absorbed energy better than a compositionally similar material organized in a planar Bouligand structure and was tougher. Finite-element modelling also showed that the sinusoidal architecture found in the impact region of the smashing appendage redistributed stress better than the classical Bouligand structure [33].

Another aspect of the mantis shrimp smashing appendage applied to biomimetics has less to do with fibre organization. The impact region of the appendage is covered by a layer of small apatite particles in a fibrous organic matrix that penetrates the particles. This material demonstrably increases the impact resistance of the surface. The coating effectively dissipates energy via the breaking and the amorphization of the nanoparticles, while the organic matrix contributes damping [73]. Surface modifications do not relate only to fracture resistance, but may affect other material characteristics, such as wear resistance and wettability. Both effects have been observed in isopods. The sliding surfaces in their leg joints may be structured, resulting in pairs of structured and smooth surfaces sliding against each other. In this way, wear may be reduced [5]. The structuring of the outermost layer of the cuticle in terrestrial isopods affects the wettability of the body surface, which may result in self-cleaning and the prevention of adhesion [74]. These aspects of the skeleton have biomimetic potential as well.

Biomimetics can also be taken beyond material properties to the design of larger structures. Inspired by the skeleton of the lobster, ICD/ITKE Research Pavilion 2012 designed by the University of Stuttgart incorporated a variety of crustacean skeletal features, with helicoidally arranged fibres resulting in isotropic material properties as well as fibres aligned in the direction of loading. The structure was constructed from a composite of carbon fibres and a synthetic resin, deposited by robotic winding of fibres over a temporary scaffold. The resulting pavilion measured 8 m across but weighed only 320 kg and its walls averaged only 4 mm in thickness [75].

## 11. Recent and future advances

Recently, research on crustacean exoskeletons has shifted towards more complex skeletal structures than the tergites and the carapace, particularly those that are subjected to great forces. Increasing attention is given to the mineral form, the orientation of fibres and crystals, and mineral texture. This has opened up completely new ways of looking at crustacean skeletal structure and mimicking it.

When analysing the crustacean cuticle as a material, it is essential to take into account the different dimensions of cuticular properties: its mineral composition, organic fibre form and orientation, and sclerotization. Focusing on them separately cannot provide a comprehensive interpretation of how the skeleton behaves mechanically, and why. Furthermore, structural and mechanical characteristics, biological processes of cuticle formation and biological functions of skeletal elements should be considered integratively as much as possible when studying crustacean skeletons. Importantly, hypotheses about putative functions should be discussed, but not taken as fact without testing. While fibre orientation and combinations of materials with differing elastic moduli have received attention and inspired applications, future developments might consider combining different aspects of cuticular structure. Crustaceans modify a great range of skeletal properties that combine to establish the outstanding performance of their skeletons. The more of these material property dimensions are considered in biomimetic designs, the greater their potential. To this end, good communication between biologists and material scientist is essential, as each of them can contribute insight into different aspects of skeletal research.

We still need to learn a lot about how the cuticle is deposited. The current view of this process is that chitin–protein fibres self-assemble some distance from the epithelium during cuticular deposition and that the change in angle between sequential planes depends on the proteins associated with them [6]. However, there is opportunity to modify this model to account for differences in lamellar thickness between body parts and during growth, as well as the deposition of parallel fibres. Furthermore, much remains to be determined regarding how complex cuticular structures are deposited by epidermal cells. The processes of sclerotization in crustaceans are also in need of further research.

The mantis shrimp example demonstrates that the structural properties of impact-resistant crustacean cuticle can be successfully applied to biomimetic materials with improved impact resistance. We can speculate that similar success may be achieved by applying the alternative crustacean solution to impact resistance that employs flexible surface layers [53].

Cases in which deviations from the Bouligand pattern, calcium carbonate mineralization and classical three-layered cuticular structure occur are likely to take our understanding of crustacean skeletal mechanics, as well as biomimetic applications of this understanding, to a new level in the future. A great part of what is known about crustacean skeletons has been studied in the Malacostraca. Numerous other crustacean groups with dramatically different bodies and lifestyles offer opportunities for further research. If we consider the vast diversity of crustaceans and their lifestyles and the continuous development of new methods to study their cuticles, studies of crustacean skeletons and the transfer of their findings to biomimetic applications can have a bright future.

## Data Availability

This article has no additional data.
